# Eosinophil extracellular vesicles and DNA traps in allergic inflammation

**DOI:** 10.3389/falgy.2024.1448007

**Published:** 2024-08-01

**Authors:** Tobias Weihrauch, Rossana C. N. Melo, Natalie Gray, David Voehringer, Peter F. Weller, Ulrike Raap

**Affiliations:** ^1^ Division of Experimental Allergy and Immunodermatology, Faculty of Medicine and Health Sciences, Carl von Ossietzky University Oldenburg, Oldenburg, Germany; ^2^ Laboratory of Cellular Biology, Department of Biology, Institute of Biological Sciences (ICB), Federal University of Juiz de Fora, UFJF, Juiz de Fora, Brazil; ^3^ Division of Anatomy, Faculty of Medicine and Health Sciences, Carl von Ossietzky University Oldenburg, Oldenburg, Germany; ^4^ Department of Infection Biology, University Hospital Erlangen, Erlangen, Germany; ^5^ FAU Profile Center Immunomedicine (FAU I-MED), Friedrich-Alexander Universität Erlangen-Nürnberg, Erlangen, Germany; ^6^ Division of Allergy and Inflammation, Department of Medicine, Beth Israel Deaconess Medical Center and Harvard Medical School, Boston, MA, United States; ^7^ Research Center for Neurosensory Science, Carl von Ossietzky University Oldenburg, Oldenburg, Germany; ^8^ University Clinic of Dermatology and Allergy, Carl von Ossietzky University Oldenburg, Oldenburg, Germany

**Keywords:** extracellular vesicles, eosinophil activation, sombrero vesicles, EETosis, inflammation, eosinophilic diseases

## Abstract

Eosinophil granulocytes, a specialized subset of white blood cells, have traditionally been associated with allergic responses and parasitic infections. However, recent research has unveiled their versatile roles in immune regulation beyond these classical functions. This review highlights the emerging field of eosinophil biology, with a particular focus on their release of extracellular vesicles (EVs) and extracellular DNA traps (EETs). It further explores potential implications of eosinophil-derived EVs and EETs for immune responses during inflammatory diseases. The release of EVs/EETs from eosinophils, which also affects the eosinophils themselves, may influence both local and systemic immune reactions, affecting the pathophysiology of conditions such as airway inflammation, chronic rhinosinusitis and atopic dermatitis.

## 1 Introduction

Eosinophil granulocytes are innate immune cells which compromise 2%–10% of all circulating leukocytes (1). They were first described by Paul Ehrlich in 1879 due to their bright color when stained with the acidic dye eosin (2, 3). Human eosinophils have a diameter of 12–15 µm, bilobed nuclei, and a lifespan of up to 18 h in the peripheral blood (4, 5). Once eosinophils have entered into tissues, they normally do not recirculate and have a life span of two to five days (6).

Even though eosinophils play an increasing role in allergic inflammation, they were originally considered to be a part of host defense against helminths and extracellular bacteria. When opposing these infections, eosinophils can release reactive oxygen species and toxic granule proteins such as major basic protein (MBP), eosinophil-derived neurotoxin (EDN), eosinophil peroxidase (EPX), and eosinophil cationic protein (ECP) (7). Further, extracellular DNA traps (ETs) are formed to bind and kill bacteria (7–12). Eosinophil extracellular traps (EETs) are not only associated with host defense but also with several inflammatory diseases and potentially contribute to disease persistence and progression (12, 13).

Eosinophils store and secrete a variety of inflammatory mediators such as interleukin (IL)-4, IL-5, IL-13, IL-31, neurotrophins like nerve growth factor (NGF), and brain-derived neurotrophic factor (BDNF) (10, 14–16). Furthermore, eosinophils can release different types of nanoscale membrane-bound structures, collectively termed extracellular vesicles (EVs), which have the ability to carry specific immune mediators, thus denoting the complexity of eosinophil secretory mechanisms. In human eosinophils, endosome-derived exosomes, plasma membrane-derived microvesicles/MVs, and secretory granule-derived eosinophil sombrero vesicles/EoSVs can act as EVs which are associated with inflammatory responses (17, 18). Besides eosinophils, other cells such as dendritic cells, macrophages, monocytes, neutrophils, and T cells also release EVs, contributing to inflammatory diseases such as asthma and atopic dermatitis (AD) (19, 20). This review aims to give an overview on eosinophil EVs and EETs and their contribution to inflammation.

## 2 Eosinophil sombrero vesicles in inflammation

**Figure 1:**
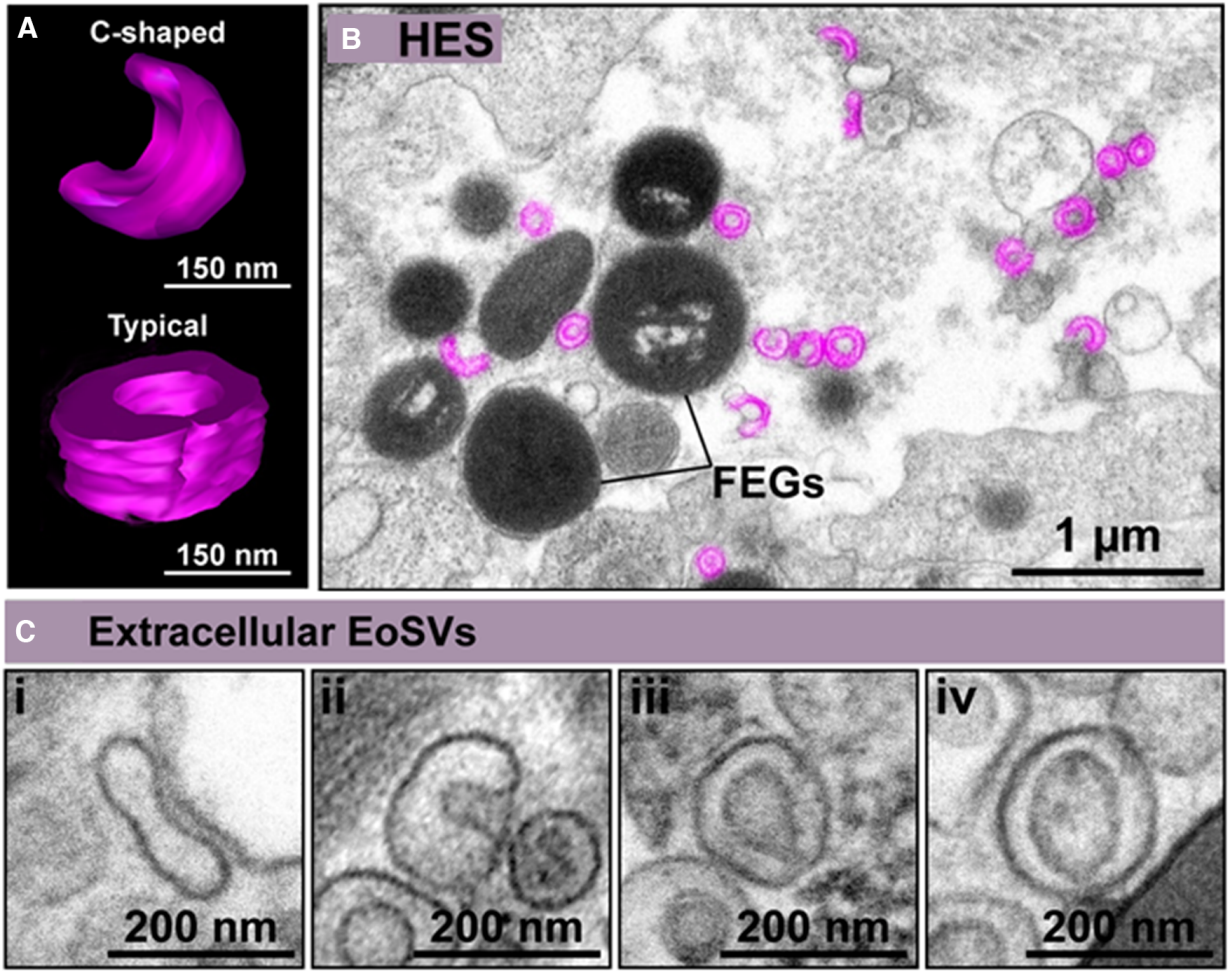
Extracellular eosinophil sombrero vesicles (eoSVs) are hallmarks of eosinophilic inflammation. (**A**) C-shaped and typical EoSVs are shown in three dimensions (3D) by electron tomography. (**B**) Representative electron micrograph of hypereosinophilic syndrome (HES) skin shows free EoSVs (colored in pink) in proximity to free extracellular granules (FEGs). (**C**) High magnification of extracellular tissue EoSVs with intact membranes and elongated, C-shaped, and typical morphologies. Reprinted from Neves et al., 2024 (26) under a Creative Commons Attribution 4.0 International (CC BY 4.0).

Named as “microgranules” or “small granules” in the earlier eosinophil literature, EoSVs are large vesicular-tubular carriers, which are present in the cytoplasm of human eosinophils and involved in the transport of immune mediators (21–25). Due to their typical morphology, EoSVs are easily identified by transmission electron microscopy (TEM) (24, 25). EoSVs have a diameter of 150–300 nm (22) and were named due to their vesicular-tubular structure and their resemblance to a hat, although they can also take on an elongated or “C” shaped morphology (Figures 1A–C) (21, 26).

Remarkably, EoSVs are associated with eosinophil activation both *in vivo* and *in vitro* (27). Conventional TEM and 3D electron tomography studies have demonstrated that EoSVs are found around, and attached to secretory granules in the cytoplasm of activated eosinophils (21). This is because EoSVs can be formed from granules and are actively trafficking granule-derived products such as cytokines and cationic proteins (21). The total number of EoSVs increases when eosinophils are exposed to inflammatory stimuli. Furthermore, the number of EoSVs in contact with secretory granules also increases, thus influencing the release of the EoSV content into the extracellular medium (21). Moreover, in hypereosinophilic syndrome (HES) patients, who present with naturally activated eosinophils, the number of EoSVs is significantly higher than in healthy individuals (23).

EoSVs carry products from secretory granules to the cell surface, enabling the rapid release of specific mediators (21). This process of eosinophil secretion based on vesicular transport is termed piecemeal degranulation (PMD) and is frequently observed *in vivo* during numerous diseases (28). PMD is involved in the release of major basic protein (MBP) by EoSVs, which can be observed after stimulating eosinophils with C-C motif chemokine ligand 11 (CCL11). Moreover, it has been reported that the Th1 cytokine tumor necrosis factor (TNF-α) induces the release of granule contents through compound exocytosis (29). Both CCL11 and TNF-α lead to the increase of the number of EoSVs in the cytoplasm of eosinophils. Another experiment showed that these cell-activating stimuli also cause a threefold amplification of EoSVs that carry interferon-γ (IFNγ) (29). IFNγ-positive EoSVs are mostly found in the peripheral cytoplasm rather than in the adjacent cytoplasmic area deeper in the cell, suggesting a fast response to inflammatory stimuli (29). Melo et al. also demonstrated that both EoSVs and small spherical vesicles transport IL-4, a cytokine that plays a major role in atopic diseases, from specific granules to the plasma membrane upon CCL11 stimulation (22). Furthermore, it has been suggested that in human eosinophils, transferrin receptor-positive (TfnRc) recycling endosomes play an important role in trafficking the Th2 cytokines IL-9 and IL-13 to the plasma membrane for subsequent release (30).

Interestingly, cell-free EoSVs (delimited by their bilayer membranes) have also been identified in inflamed tissues of different target organs involved in eosinophilic diseases such ulcerative colitis (13, 26), eosinophilic chronic rhinosinusitis (ECRS) (13, 26), HES (Figure 1B) (26), dermatitis (26), schistosomiasis mansoni (26), and eosinophilic esophagitis (31). Intact free EoSVs are released as a result of eosinophil cytolytic degranulation and remain in the extracellular matrix in conjunction with free extracellular granules (FEGs) even after complete cell disintegration except for granules (26). For eosinophils, cytolysis including cytolysis with the release of nucleus-derived EETs (EETosis), is a form of eosinophil degranulation commonly observed *in vivo* (26, 32, 33). Extracellular EoSVs are mostly observed close to FEGs, but also in the proximity of EETs (26). Increased evidence suggests that the release of EETosis-generated EETs plays a crucial role in the pathophysiology of inflammatory diseases, with the resulting release of both intact FEGs and EoSVs being involved in eosinophil responses (26).

## 3 Eosinophil-derived microvesicles and exosomes in inflammation

Eosinophils release high numbers of MVs upon stimulation with inflammatory stimuli as shown in a study by Akuthota et al. (17). When eosinophils were exposed to CCL11 or TNF-α, the release of MVs was significantly increased when compared to unstimulated eosinophils. MVs, which by definition are EVs directly formed from the plasma membrane, were captured *in situ* and quantitated with the application of TEM, which unambiguously enables visualization of EVs at the cell surface (17, 18). The authors demonstrated that the type of stimulus modulates both the production and size of MVs. TNF-α resulted in greater secretion of MVs compared to CCL11, and, on average, these TNF-α-induced EVs had smaller diameters, which possibly reflects an effect of faster EV production on membrane dynamics (12). Interestingly, CD9 proved to be a better marker for eosinophil-derived MVs than CD63. This was revealed by both immunogold transmission electron microscopy (TEM) and nanoscale flow cytometry. This is in accordance with the fact that the surface of human eosinophils constitutively express high levels of CD9 (34).

Exosomes have been detected in eosinophils from patients with asthma as shown in study by Canas et al. (35). In their study, the number of exosomes from eosinophils in peripheral blood was found to be significantly increased in the asthma group when compared to healthy controls (35). Moreover, common proteins in eosinophil exosomes such as ECP, EPX, and MBP, were analyzed for both experimental groups, as well as proteins related to migration, inflammation, or adhesion. For this, eosinophils from peripheral blood were cultured with exosomes and their migration and adhesion was investigated. Eosinophils from asthma patients exhibited increased migration activity compared to eosinophils without exosome stimuli (35). Interestingly, enhanced adhesion of eosinophils was observed only after stimulation with exosomes from eosinophils of asthmatics, but not with those from healthy controls. Particularly intercellular adhesion molecule 1 (ICAM-1) and integrin α_2_ were responsible for adhesion, as their expression was increased in the presence of exosomes (35). Furthermore, exosomes of eosinophils were shown to induce the release of reactive oxygen species (ROS) from other eosinophils as a paracrine effect. Moreover, it was observed that eosinophils absorb exosomes from other eosinophils, which has been suggested to be an energy-dependent process (35). Interestingly, eosinophil-derived exosomes from atopic and non-atopic asthmatics also have effects on other cell types. Small airway epithelial cells showed higher apoptosis rates after stimulation with eosinophil-derived exosomes from asthma patients, but not after stimulation with exosomes from healthy controls (36). The capacity of epithelial cell monolayers to repair wounds was also impaired after exposure to asthmatic exosomes (36). Lässer et al. demonstrated that EVs can be isolated directly from lung tissue. In a model of eosinophilic airway inflammation mice were sensitized and then challenged with ovalbumin (OVA) to investigate proteins from EVs in lung tissue (37). The total numbers of eosinophils in bronchoalveolar lavage fluid (BALF), EVs, and the amount of proteins were significantly increased after sensitized mice were challenged intranasally with OVA compared to those that were treated with phosphate buffered saline (PBS) (37). The enriched proteins were mostly associated with B cell signaling pathways, innate immune responses, or immune system processes such as matrix metalloproteinase (MMP-12), pendrin, arachidonate 15-lipoxygenase (ALOX15), C-C chemokine receptor type 3 (CCR3/CD193), EPX, and eosinophil cationic protein (ECP) (37). These findings suggest that eosinophils play a crucial role in airway inflammation through the release of EVs. The association of eosinophils with Th2-driven immune responses and atopy also supports the hypothesis of eosinophil derived vesicle contribution to inflammation in other atopic disorders such as atopic dermatitis.

## 4 Eosinophil extracellular DNA traps in airway inflammation

**Figure 2:**
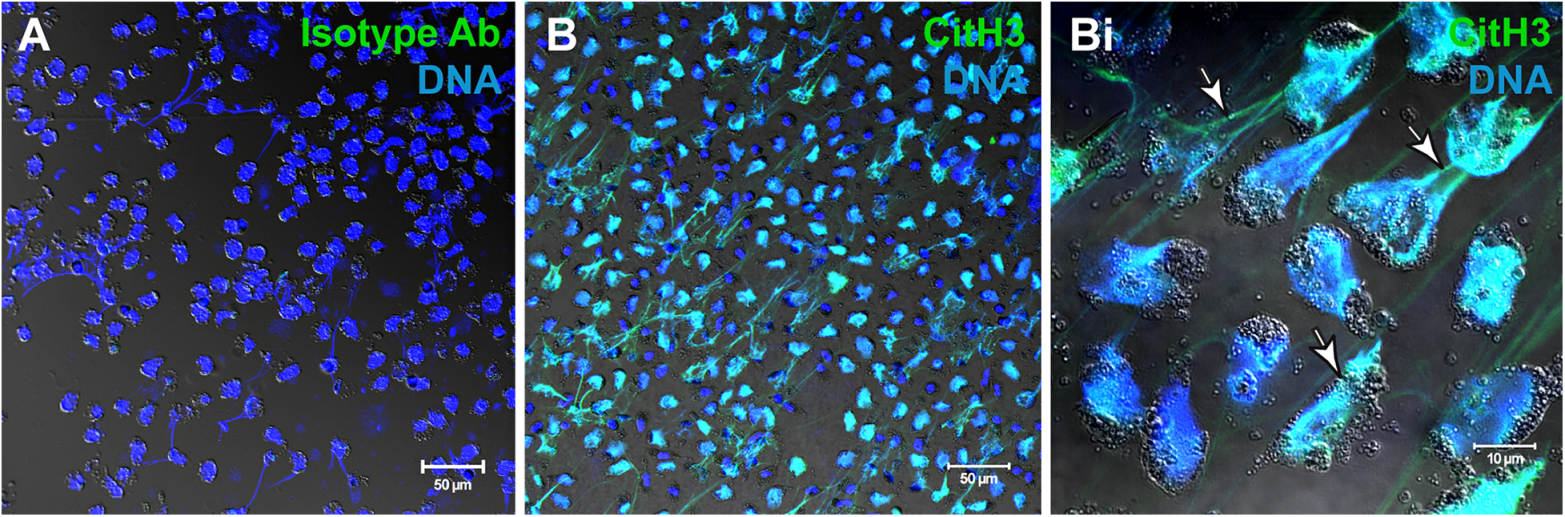
Human eosinophils undergo EETosis in response to stimulation with agonists. (**A**,**B**) Immunofluorescence for isotype (**A**) or anti-citrullinated H3 histone (CitH3) antibodies. Eosinophil extracellular traps (EETs) appear as filamentous chromatin structures immunolabeled for CitH3 (green) and DNA (Hoechst, blue). (Bi) Merged images of CitH3 +DNA-stained eosinophils in higher magnification. Arrows indicate EETs. Differential interference contrast (DIC) images were obtained by confocal microscopy. Purified eosinophils were stimulated with platelet activating factor (PAF) + IL-5. Ab, antibody. Images were reprinted from Neves et al., 2022 (13) under a Creative Commons Attribution 4.0 International (CC BY 4.0) license.

As noted, eosinophils are able to release not only different subtypes of EVs but also EETs both *in vitro* (Figure 2) and *in vivo*. ETs are not only formed by eosinophils, but also by neutrophils, the so-called neutrophil extracellular traps (NETs) and other innate immune cells such as macrophages/monocytes, mast cells, basophils, and dendritic cells (38). EETs consist of DNA fibers which are embedded with granule proteins such as MBP and ECP (12), or associated with intact FEGs (23) and EoSVs (15). The release of EETs has been described from both living eosinophils and those undergoing cell lysis (EETosis), which is dependent on nicotinamide adenine dinucleotide phosphate (NADPH) oxidase activity. The process of EET release has been suggested to be influenced by external stimuli, such as *Staphylococcus aureus*, and also depends on the time of exposure (12). Moreover, as described above, EETosis drives the release of EETs in tissues and the secretion during several inflammatory diseases (13). The formation of EETs is associated with the formation of Charcot-Leyden crystals which are composed of the protein galectin-10, a biomarker of eosinophil involvement in asthma, allergic rhinitis, and other eosinophilic inflammation (39, 13). A recent paper focusing on inflammatory eosinophilic diseases identified that early nuclear signs of EETosis appear, before the formation of nucleus-extruded EETs. This can be helpful to detect eosinophils entering in a program of EETosis *in vivo* (13).

EETs are known to play a pivotal role in severe asthma. Choi et al. demonstrated that patients presenting with severe asthma have elevated EET^+^ eosinophil counts in comparison to those with non-severe asthma or healthy controls (40). EET formation can be induced by eosinophil activation through IL-5 and lipopolysaccharides (LPS) (40), but also through thymic stromal lymphopoietin (TSLP) (41). It has however also been shown that EETs have an autocrine effect, as they induce cell activation and the release of further EETs from eosinophils (42). The formation of EETs is dependent on adhesion (41) and ROS, as demonstrated by the fact that ROS production from peripheral blood eosinophils is inhibited by N-acetyl-L-cysteine and diphenylene iodonium chloride (41, 42). This is supported by the positive correlation of EET formation and ROS production (42). Furthermore, it has been shown that the number of EET^+^ eosinophils correlates with the number of type 2 innate lymphoid cells (ILC2) in severe asthma, which is also in accordance with elevated levels of IL-13, IL-33, and TSLP plasma levels (40). These findings have been confirmed by mouse experiments in which intranasally injected fluorescence-labeled EETs were found to accumulate in lung tissue. The EET treatment caused significantly increased numbers of eosinophils and neutrophils in BALF, as well as increased epithelial thickness and higher levels of infiltration into the epithelium (40). Moreover, the BALF contained elevated levels of the epithelium-derived cytokines IL1α, IL-1β, IL-33, TSLP, and the chemokine C-X-C motif ligand 1 (CXCL-1), and CCL24. Further, the percentage of IL-5 and IL-13 releasing ILC2s was increased in EET treated mice (40). These results suggest that in severe asthma, type 2 immune responses are induced through EETs.

## 5 EETs are associated with *S. aureus* colonization in chronic rhinosinusitis

The role of EETs has also been investigated in chronic rhinosinusitis with nasal polyps (43). The presence of EET releasing eosinophils was observed in the subepithelial regions of the nasal mucosa in patients but not in healthy controls. The eosinophils were mostly found in clusters with other eosinophils, rather than being present as single EET-releasing cells (43). Interestingly, the percentages of EET-releasing eosinophils were associated with higher rates of *S. aureus* colonization which is known to be linked to increased IL-5 levels (43, 44). Furthermore, it has been demonstrated that *in vitro* exposure of tissue fragments from patients with chronic rhinosinusitis to *S. aureus*, increased the formation of EETs in eosinophils in the subepithelial regions of nasal mucosa (43). Eosinophils further migrate to sites of epithelial defects and trap *S. aureus* as shown in an *ex vivo* model of diseased human mucosal tissue. EET formation was even enhanced when isolated eosinophils from nasal secretions were stimulated with *S. aureus* (43). The release of EETs in response to *S. aureus* is also dependent on ROS production (43). Myiabe et al. impressively demonstrated the direct role of EETs in ECRS and their therapeutic potential. They showed that the presence of EETs is linked to disease pathophysiology (45). The mucus of patients was found to be highly viscous due to the presence of aggregated cells with accumulated ETs and cell debris. The treatment with EET-degrading DNase I and Heparin caused a significant decrease in mucus viscosity which is associated with a better quality of life (45).

## 6 EET link to *S. aureus* colonization indicates EET contribution to skin inflammation

The link between the release of EETs and *S. aureus* further suggests a possible role of eosinophil traps in inflamed skin such as in AD. Even though EETs could not be detected in tissue samples of AD skin in first experiments, they have been found in skin diseases related to exogenous triggers such as allergic contact dermatitis, drug hypersensitivity, and also after atopy patch tests which resemble the acute exacerbation observed in AD (46). Exposure to *S. aureus* induces itch which in turn incites scratching behavior and resulting epicutaneous skin damage, and is known to promote skin inflammation that correlates with AD severity (47–49). It has been demonstrated that IL-5 gene expression is increased in ILC2s and that IL-5 protein levels are elevated after stimulation with *S. aureus* lysate in nasal tissue of patients with chronic rhinosinusitis (50). These findings lead to the hypothesis that in inflamed skin, elevated IL-5 levels as a result of *S. aureus* colonization, induces the release of EETs from eosinophils. This assumption is supported by increased IL-5 expression in skin, and higher IL-5 serum levels in AD patients (51, 52). Furthermore, IL-5 was found to be expressed under conditions of EET formation as found in allergic contact dermatitis (46). Interestingly, NETs are released in the skin in response to *S. aureus* and have been observed to enhance colonization of this pathogen on human and mouse skin by inducing oxidative stress and the expression of the high-mobility-group-protein B1 (HMGB1) from keratinocytes (53). HMGB1 in turn promotes skin barrier dysfunctions by downregulating epidermal barrier genes, further promoting the spread of *S. aureus* (53). The microRNA-223 has been further reported to be reduced in AD patients, enhancing the release of NETs from neutrophils in AD (54). All these findings suggest that EETs might have a similar role to NETs in skin inflammation such as AD.

## 7 EVs and EETs in autoimmune disease bullous pemphigoid

EETs have further been found in skin biopsies of patients with the autoimmune blistering disease bullous pemphigoid (BP) (46) in which EVs also play a pivotal role. EVs have been reported to carry tissue-specific autoantigens in autoimmune diseases (55, 10). In a recent pilot study, BP180, a hemidesmosomal protein and the most important autoantigen in BP, was found in EVs of blister fluid in 50% of all BP patients (56). Furthermore, eosinophils have been described to be the major cellular component of BP blister fluid (57). Blister fluid exosomes from BP patients have been reported to induce the recruitment of neutrophils through IL-8 (58). It has been further hypothesized that keratinocytes and skin infiltrating granulocytes such as neutrophils, are a possible source of exosomes in BP blister fluids (58, 10). As enhanced release of TNF-α from keratinocytes has been observed after stimulation with blister fluid derived exosomes (58), it is possible that TNF-α in turn initiates the release of EoSVs from eosinophils, contributing to the pathogenesis of BP.

## 8 Final remarks and questions for the future

In summary, increasing evidence has been demonstrating that eosinophil EVs and EETs play a pivotal role in inflammation during a variety of diseases associated with eosinophilia, including in the upper and lower airways and the skin. However, the question arises if there is there also a marker for eosinophilic diseases as a result of EET release. A promising candidate is Galectin-10, as it is released in large amounts by EETosis rather than the secretory system (59). Furthermore, the role of ETs in mucus and tissues of patients with eosinophilic diseases poses another question that has not yet been answered. Further research is required to answer these questions and to gain a comprehensive understanding of EETs and EVs in eosinophilic inflammatory diseases, which may lead to novel and therapeutic treatment approaches in the future.
